# Getting through the day: a pilot qualitative study of U.S. women’s experiences making decisions about anti-nausea medication during pregnancy

**DOI:** 10.1186/s12884-018-2093-6

**Published:** 2018-12-04

**Authors:** Marlaine Figueroa Gray, Clarissa Hsu, Linda Kiel, Sascha Dublin

**Affiliations:** 1 Kaiser Permanente Washington Health Research Institute, Seattle, WA USA; 2 University of Washington Epidemiology Department, Seattle, WA USA

**Keywords:** Pregnancy, Nausea, Vomiting, Medication, Anti-emetic, Ondansetron, Qualitative, Focus group, Decision making

## Abstract

**Background:**

Nausea during pregnancy affects 80% of pregnant women and can severely affect women’s functioning and quality of life. Women often have difficulty deciding whether to take anti-nausea medications due to concern about medication risks. This paper foregrounds U.S. women’s voices as they share their experiences making decisions about anti-nausea medication use.

**Methods:**

As a pilot study, we conducted two focus groups including 20 women who had filled at least one prescription for an anti-nausea medication during pregnancy. Topics included deciding about and taking anti-nausea medications. Transcripts were analyzed by two medical anthropologists using an inductive or open coding approach.

**Results:**

Women in our pilot study carefully considered whether to take anti-nausea medications. Most women preferred not to take medications, in general, but were willing to do so for severe symptoms. When considering medications, they expressed concerns about risks to fetal health. They considered information from internet research, their health care provider, and the experiences of friends and family. While some women in our study decided against taking medications, many did take a prescription medication, and they reported substantial improvement in their symptoms and sense of well-being.

**Conclusions:**

Women weighed various sources of evidence to assess the risks and benefits of taking anti-nausea medication and ultimately made a range of choices. More research is needed about the effectiveness and risks of anti-nausea medication, to help support women in their decision-making process, and also about the best methods to communicate scientific evidence to women.

**Electronic supplementary material:**

The online version of this article (10.1186/s12884-018-2093-6) contains supplementary material, which is available to authorized users.

## Introduction

About 80% of pregnant women experience nausea and/or vomiting during pregnancy—over 3 million women each year in the US [1, 2]. These symptoms can profoundly affect women’s lives. Consequences can include decreased ability to function at work and at home; depression; concern for the health of the baby; emergency department visits and hospitalizations; and significant economic burden [3–8]. Many women consider taking a medication for their symptoms. Yet this decision can be difficult because there is little evidence about the safety or effectiveness of most medications that are currently used to treat nausea and vomiting in pregnancy [9], as was concluded by a recent Cochrane review. Only one medication, Diclegis (doxylamine/pyridoxine) is currently approved by the US Food and Drug Administration (FDA) to treat nausea and vomiting during pregnancy; it is very expensive and often not covered by insurance. The most commonly used medication in the US, ondansetron, is not FDA-approved, but is currently used by about one in four pregnant women. Its safety remains controversial. Few relevant randomized clinical trials have been conducted, and most were very small and had very short follow up [9]. Thus, most of the evidence about these medications’ safety currently comes from retrospective epidemiologic studies. Two epidemiologic studies have suggested that ondansetron may increase the risk of birth defects [10, 11]; one found a higher risk of cleft lip/palate [10] while the other found higher risk of heart defects [11]. A third study found no association overall [12] but lacked power to examine individual birth defects. The findings of these studies have been questioned [13], and the role of ondansetron in treatment remains somewhat controversial [13–15]. Overall, because of inadequate evidence about medication safety in pregnancy [9], women with nausea and vomiting face a dilemma when making decisions about treatment. Little is known about women’s perspectives and decision-making process when confronting this difficult decision.

Several studies have examined women’s experiences with nausea and vomiting in pregnancy, more broadly [16–20]. One study conducted focus groups with women and clinicians in Norway and found that women felt their experiences of nausea and vomiting were not taken seriously [17]. Pregnant women and clinicians were reluctant to consider medication use because of concerns about harm to the baby. In Norway, women and clinicians relied heavily on the option of graded sick leave (part-time work), which is less available in the US. A study in Canada interviewed women calling a telephone counseling line to learn about treatment options for nausea and vomiting in pregnancy; the authors found that despite detailed counseling about Diclegis (doxylamine and pyridoxine), a medication widely regarded as safe in pregnancy, many women remained fearful and continued to believe it caused birth defects [16]. This study took a quantitative approach, reporting survey results but not foregrounding women’s voices or providing details of their experience. Finally, Locock et al. conducted qualitative interviews with 73 women from the UK [18]. While this paper provides a rich and detailed view of women’s experiences with nausea and vomiting more broadly, little information was provided about women’s opinions and choices regarding medication use. No study has examined the perspectives of women in the US. The only study about women’s perspectives came from Norway [17], where attitudes and practices may be very different from the US because of greater access to paid sick leave and part-time work. This paper will examine the perspectives of U.S. women who chose to fill an anti-nausea medication prescription to understand how they made decisions about whether to take anti-nausea medication during pregnancy, including their attitudes regarding medication use, how they weighed risks, what evidence they considered, and their experiences using anti-nausea medication.

## Methods

### Overview

This pilot study was set within Kaiser Permanente Washington (KPWA), an integrated healthcare system in the northwestern United States with about 710,000 members and about 6000 births per year. Our study team conducted two focus groups with a total of twenty women who had filled at least one prescription for an anti-nausea medication during pregnancy. Our reporting of participant sampling, data collection, and data analysis follows the consolidated criteria for reporting qualitative research (COREQ) guidelines [21].

### Sampling/recruitment

We used purposive sampling that focused on recruiting women living in the Seattle/Tacoma metropolitan area who were 18 years and older, who delivered a liveborn infant in 2015 or 2016, and who had filled at least one prescription for an anti-nausea medication during pregnancy (Table 1). Filling at least one prescription indicated to us that women experienced enough of a burden from nausea that they communicated with their provider about their symptoms and were likely to have actively considered the pros and cons of taking anti-nausea medication. Purposive sampling involves identifying individuals that have knowledge or experience with a phenomenon of interest and are willing to participate in research [22]. We excluded women with other serious health conditions during pregnancy (pregestational diabetes, cardiac disease, renal disease, liver disease, autoimmune disease, HIV, or a major psychiatric disorder) or adverse pregnancy outcomes such as stillbirth or major birth defects. KPWA electronic health records were used to identify women meeting these inclusion and exclusion criteria.

**Table 1:** Participant Characteristics

Characteristic		Number (*N* = 20)	Percent
Age	18–24	2	10%
	25–29	5	25%
	30–34	5	25%
	35–39	7	35%
	40–44	1	5%
Medication used for nausea during pregnancy	Any use of anti-nausea medication	18	90%
	Any use of Ondansetron	12	60%
	Any use of medication other than Ondansetron	4	20%
	Don’t Know/Can’t Remember which medication was taken	2	10%
	No medication used	2	10%
Employment	Full-time	14	70%
	Part-time	1	5%
	In school/vocational training	2	10%
	Homemaker	3	15%
Main Provider of Pregnancy Care	Obstetrician	11	55%
	Family Practitioner	3	15%
	Midwife	6	30%
Marital Status	Married/living with partner	18	90%
	Not married/not living with partner	2	10%
Education	Some HS^a^, not a graduate	1	5%
	HS^a^ graduate or GED^b^	1	5%
	Some college/2 year degree	4	20%
	Four-year degree	6	30%
	More than four year degree	8	40%
Race	White/Caucasian	13	65%
	Black/African American	1	5%
	Asian	3	15%
	Native Hawaii/Pacific Islander	1	5%
	More than one race	2	10%
Ethnicity	Hispanic or Latina	0	0%
	Not Hispanic or Latina	20	100%
Home Ownership	Own	12	60%
	Rent	7	35%
	Living with friends/extended family	1	5%
Number of births	1	8	40%
	2	10	50%
	3	1	5%
	Missing	1	5%

^a^high school

^b^General Educational Development (a series of tests available in the US that provide certification that the test taker has high school level academic skills)

We then mailed a recruitment letter to a random sample of 700 women from the eligible population. Women could call in to volunteer for focus groups. Within a week of sending the recruitment letters we began reaching out to potential participants by phone to invite them to participate and continued these calls until focus groups had been filled. In all, 20 women attended the focus groups.

### Data collection

The focus group guide (themes represented by Table 2, and full guide available by supplemental attachment) was developed through an iterative process. We approached the design of the interview guide using a phenomenological perspective, which focuses on developing rich descriptions of lived experiences, in this case women’s experiences making decisions about medication use during pregnancy. The principal investigator (SD), an internist and pharmacoepidemiologist, drafted questions to elicit how women make decisions about medication use during pregnancy, and two team members who have doctorates in medical anthropology (MFG and CH) developed the guide with input from the entire team. Because this was a small pilot study, the guide was not pilot tested before use with our participants. The full.

**Table 2:** Focus Group Questions/Themes – see Additional file 1 for complete focus group guide

Question Type	Examples
Building Rapport	What you would like us to call you? How many children do you have?
Questions about women’s experience with nausea or vomiting in pregnancy.	We would like to go around and have you briefly describe how nausea during pregnancy affected your life.
Questions about women’s experience making decisions about how to treat nausea and vomiting in pregnancy.	Did your doctor or midwife suggest any treatments? Did he or she offer to prescribe medications to treat your nausea/vomiting? (Vit B6, Unisom) What was the most important thing that you considered when deciding whether to take a medicine for nausea and/or vomiting in pregnancy?
Questions about women’s experiences with medications to treat nausea and vomiting in pregnancy	Now let’s talk about your experience with medications. For those of you who took medication for nausea, can you describe your experience with medications? [*Note –ask probing questions for each medication*].For this kind of medication, what does effectiveness look like to you? For example, how would you know if an anti-nausea medication was working?

A team member with advanced qualitative expertise (MFG) led the focus groups. Another team member with advanced qualitative expertise (CH) assisted with consenting, logistics, and scribing. A court reporter transcribed each 90-min discussion, and each discussion was also audio-recorded. Participants met each other and the facilitators for the first time when they arrived at the conference room in a KPWA clinic. Participants were given table tents and assigned a number which was used by the court reporter during transcription. Participants were asked a semi-structured series of questions (Table 2), and the facilitator asked probing questions to draw out topics of interest and clarify respondents’ comments. Participants responded to questions round-robin style in the beginning, but by the second question organic conversation developed. Transcripts were checked against the recording.

### Data analysis

We used an inductive analysis approach [23], including an iterative code development process. The primary coder (MFG) drafted an initial “first cycle” code list [24]. Two team members (MFG, CH) then coded and compared one transcript. Codes were revised and code definitions and domains were clarified. The primary coder then reviewed and revised the coding for both transcripts using the final version of codes and domains [24]. In this small pilot study, data saturation was not a goal and was not discussed during analysis.

Next, the full research team discussed key themes that provided insight into women’s experience deciding whether to take anti-nausea medication during pregnancy. Data were then extracted by code for all relevant codes and the first author reviewed these data again for subthemes and nuanced insights. The first author drafted a memo containing coded themes which was discussed with other team members and used to structure the findings presented in this article.

## Results

Focus groups included twenty women (10 per group). Table 2 summarizes participant characteristics. Generally, they had high socioeconomic status (SES) as indicated by their educational status and the high rate of home ownership. Similar themes arose in each group.

### Women’s general attitudes toward medication use during pregnancy

Women reported being very careful about what they consumed during pregnancy. Women described wanting to ingest only “natural” substances while pregnant and were concerned about the risks of pharmacological treatments to fetal health. They described the choice to take medication as one in which they weighed their comfort against their baby’s well-being, and the choices they made about medication use predominantly reflected what they understood as promoting the baby’s well-being. In general, women in our focus groups voiced a preference for not taking prescription medication during pregnancy, when possible.

Focus Group 1, Participant 1: With my first kid, I was so natural and everything was smooth. So I wanted to do everything I did with the first kid, so the second would come out the same…[For my severe nausea] I asked my doctor what I could do, and she prescribed me some pills. I had them in my hand but the idea that it was a prescription just freaked me out and I didn’t want to put any foreign objects in my body. I don’t know what it’s going to do to the baby. [The nausea] was really bad, but I was scared for the baby. I was just going back and forth: pills, baby. [Participant makes hand motion to demonstrate weighing two options].

Focus Group 1, Participant 2: Just like a lot of you, I don’t like to be on medication either. Actually, this was the first time I’ve ever actually taken medication being pregnant.

### Pathways to prescription

While participants were selected into our focus group because they had all filled a prescription for anti-nausea medication, their pathways to getting a prescription varied. Women who participated in our study described experiencing nausea during their first trimester, during which time prenatal visits with providers occur less frequently than later in pregnancy. We learned that decision-making about taking anti-nausea medication began before being given a prescription as women made choices about scheduling early appointments to discuss nausea or ask for help in dealing with nausea. Several women visited a provider in the first trimester for nausea and vomiting and were offered a prescription. Some women requested a prescription as a result of the severity of their symptoms and/or recommendations from friends and family members. Providers often initially recommended non-prescription treatment options such as over the counter vitamin B12 (pyridoxine) with or without doxylamine and then provided a prescription for a medication if the initial treatment approach did not provide relief (in keeping with current clinical guidelines) [15]. The prescription medication women most often talked about using was ondansetron (brand name Zofran).

Focus Group 2, Participant 8: I asked my doctor [for the medication]. Back then, I was just worried all the time how my baby was going to survive, because I lost 15 pounds in a month.

Focus Group 2, Participant 6: I didn’t even know to ask for the medicine until my friend who’s a doctor and his wife had had twins. He suggested it.

Some of the participants did not discuss their nausea and vomiting with their providers because they thought their experience was a normal part of pregnancy. These women often did not get prescribed medication until later in their first trimester or when they ended up in the emergency department with dehydration.

Focus Group 2, Participant 10: I never mentioned my nausea to my provider originally because I assumed this is what everybody goes through. I ended up getting it prescribed at Urgent Care because it got to that point.

### Weighing risks of medication

Regardless of how they received their prescription, nearly all of the women gave serious consideration to the possible risks of taking medication. Women were primarily concerned about negative birth outcomes or long-term effects on their child’s health.

Focus Group 2, Participant 10: I did fill it and I took it the one time and I got anxious about the side effects. I’m always concerned about taking medicine when pregnant, so I did look it up and I heard about the heart [defects] but also cleft lip. I was [worried about] risks that would affect him long-term. I was very anxious about anything affecting my baby. So I just kind of figured I’d get over [the nausea].

Some women felt that their intense nausea interfered with their ability to function socially and professionally, and that this stress took a toll on their health and their baby’s health. For these women, nausea was disruptive and demoralizing. They described having to stop doing the things that they enjoyed as well as the things they needed to do such as care for their children. They thought that the benefit provided by the medication was worth the risk.

Focus Group 1, Participant 7: I felt tired all the time, and I couldn’t focus at work. And it’s not good for my health and not good for my baby and just weighing the risk and the benefit for taking the nausea pill, I just think maybe it’s better, yeah, to take the medication.

Focus group 1, Participant 6: You have to stand up all day, and you’re lifting [heavy items]. And I couldn’t take time to be sick. So I took the Ondansetron, which is what the doctor gave me and I just lived on it for, like, three months.

Focus Group 2, Participant 9: The lowest point was I was at work, so nauseous, and then I had to ask the office manager, “Do you have an empty room so I can sleep?” I laid on the floor with my jacket. It was so sad. It was really sad.

Focus group 2, Participant 7: Some days I had to miss school because I couldn’t get out of bed. I wasn’t eating. So I had to get on the pills to eat at school. Sometimes I had to go home from school because I couldn’t stomach watching everyone eat.

Focus Group 2, Participant 3: I was taking public transportation and I had to stop because every time I would be on the bus I would throw up.

Other women described not knowing when nausea and vomiting would occur and how this uncertainty limited their ability to move through their days. Treatment allowed them to regain their confidence and enjoy the experience of being pregnant.

Focus Group 1, Participant 6: To me, there was so much stress and misery with not knowing what -- if you were going to be able to go to work or do I have to call out or can I go to the grocery store? I just wanted my pregnancy to have a -- to be jovial. I just wanted, like, an overall good juju with the pregnancy, so I just wanted there to be a happy spirit the whole time. So that to me was really important.

### What evidence matters to women

We sought to understand how women weighed and interpreted different kinds of evidence. Women got their information from a variety of sources. Many sought information by reviewing the published literature or consulting websites. Others were reassured by their doctors, by the length of time the drug has been on the market or by the experiences of their friends and family.

Focus Group 1, Participant 7: I just looked up Wikipedia, and it said it was safe. I think it’s Class A or B, so it’s relatively safe. So I just decided to try it.

Focus Group 2, Participant 4: For Zofran [ondansetron], there’s few studies, a handful, about heart defects in the baby, but I think that the percentage was from 0.8% or it could have happened anyway from 0.2%, so it wasn’t that much of a difference.

Two women were concerned about the lack of research on human fetuses or involving women like them (for instance, of the same racial or ethnic background).

Focus Group 1, Participant 7: Whatever research they do they only do it on animals, not, like, real human babies. So just in the back of my mind I would always worry,

Focus Group 1, Participant 1: There wasn’t, for me, I guess, enough research that said what it would do to my body. Are they testing on women that look like me, that are my size, my shape, my ethnicity? Is it really going to help me? And that’s what -- I’m just like -- I just can’t.

Other women found their doctor’s reassurances to be sufficient evidence of safety.

Focus Group 1, Participant 6: The doctor went to school for that. That’s their job. So if they say it’s okay, it’s okay. So I didn’t feel like if they were giving me it that there would be any huge risks associated.

Some women considered the length of time the medication had been in use by pregnant women to be evidence of safety.

Focus Group 1, Participant 4: Knowing that it had a long history of pregnant women taking it helped assure me that this is probably okay. If you’ve been having something prescribed for a couple decades [any risks] probably would have come out by then.

Other women were influenced by the experiences of family, friends and acquaintances.

Focus Group 1, Participant 11: I called my sister because she was the one who told me about it. She’s like, “I’ve used it for all of my pregnancies. They’re probably just trying to make some correlation, and there’s no [correlation].” -- So I was like, “Okay. That was reassuring enough for me.”

On the other hand, one woman whose friend had a baby with a birth defect decided not to take the medication.

Focus Group 2, Participant 1: I didn’t end up taking the Zofran. One of my close girlfriends had a son [with] a clubfoot and a cleft lip because of it because she had to take so much of [the medication]. And the doctors told her it was because of that, so I was in a lot of fear of taking that.

### Medication experience

Though two women in our groups decided not to take the medication after filling the prescription, a majority (18/20 or 90%) took their prescribed anti-nausea medication at least once. Many women reported that the benefits of the medication were significant, allowing them to regain their appetite and function throughout their day.

Focus Group 1, Participant 2: My prescription was a saving grace. Once I actually took it and I was able to desire foods again, I was like, “Oh, I’m sold”. And I could literally get through the day.

Focus Group 1, Participant 4: I was like, “This is my little miracle thing. It makes it go away, and I feel like I can function way better.”

Some women considered the risks and either stopped taking the prescribed medication or never took it at all. They thought they could tolerate the physical discomfort, or they were concerned about negative effects on their child.

Focus Group 2, Participant 10: I stopped taking it because it made me more scared for any possible risk or whatever, so I couldn’t do it. So I just lived in the pain because I couldn’t get over that.

Finally, some women reported that their medication worked well, but they had to stop because of side effects such as severe constipation or hives.

## Discussion

This study is the only qualitative study that specifically focuses on pregnant women’s experiences related to taking anti-nausea medication, something millions of women around the world do each year [2]. We explored how women made the decision to take anti-nausea medications and what evidence they considered when making that decision. As was previously reported [25], we found that nausea and vomiting during pregnancy can profoundly disrupt familial, social and professional roles and impact the happiness some women expect to be associated with pregnancy. These social and professional impacts were often as or more important to women in our study than the physical impacts of nausea and vomiting, and they played a significant role in women’s decisions. Women described how symptom relief from medications allowed them to take care of their children, commute to work, attend school, and perform their professional duties. Many women in our study who in general preferred to avoid medication use during pregnancy turned to pharmacological relief in this situation.

In other studies on this topic, some women described feeling that providers continued to disbelieve the severity of women’s symptoms and did not offer adequate early care to women [17, 20]. While our study did not reveal examples of providers not offering adequate care, we did find that KPWA clinicians were cautious about prescribing medication and typically began with vitamin supplements before offering prescriptions, which is consistent with clinical guidelines from national professional societies [15]. We noted two factors – this caution around prescribing and also the practice of not scheduling routine prenatal visits until fairly late in the first trimester—influenced whether women were prescribed medication to relieve their symptoms at the time when symptoms were most severe. This manuscript brings attention to a symptom that can negatively impact women’s quality of life, ability to function, ability to maintain social and professional roles, and their sense of self. Focusing attentive concern on a symptom which is often dismissed as a normal part of pregnancy can help providers give more patient-centered care to their pregnant patients.

Our findings regarding the personal, professional and psychosocial impact of nausea and vomiting on women’s lives are consistent with previous work describing women’s experiences. Locock et al. [18] found that nausea and vomiting in pregnancy can be so severe that women experience a loss of self and “biographical disruption”, a loss of confidence in the body that leads to a loss of confidence in one’s self-identity [18, 25]. Because this disruption can be severe, women do want to find a way to maintain their sense of self-identity and often seek medication to help them manage their symptoms and return to feeling authentic and able to perform their social and professional roles. Our participants reported that symptom relief also allowed them to enjoy their pregnancy.

Other researchers have found that pregnant women face the burden of weighing various types of evidence to assess risk to personal and fetal health [26]. When women in our study considered medication use, they drew on different kinds of evidence to weigh risks and benefits. As in our study, Baggley’s survey of 59 Canadian women found that women received medication information from a variety of sources, with a large majority receiving it from family and friends [16]. Baggley also noted that many women who had received evidence-based counseling about medication safety often did not feel convinced or reassured [16]. In our study, women found that personal experiences of friends and family were very powerful assurances. Understanding what kinds of information women consider as evidence of medication safety may be helpful to clinicians as they seek to convey information to women.

### Strengths and limitations

This study has several strengths. By recruiting women who had filled a prescription for medication and asking them specifically about medication use and decision-making, our study provides a unique perspective on how women make decisions about medication use for nausea and vomiting in pregnancy, what evidence they consider, and what benefits matter to them. We recruited women from an integrated health system which represents community-based practice, so they likely are more representative of the general population compared to patients from academic practice or a tertiary care setting. As far as we know, this is the only paper, either quantitative or qualitative, that has studied the experience of women taking anti-nausea medication during pregnancy.

This study also has limitations. By its nature, qualitative work is not expected to be generalizable to larger populations. We talked with a relatively small number of women who were predominantly white, married, and well educated. They might have had better access to resources to help them manage their nausea, as well as more flexibility in their work and care giving duties. Women with fewer resources might experience an even greater burden in managing the effects of their symptoms. There may be geographic variation in perspectives and experiences that we were not able to examine. Because this was a pilot study, we did not continue holding focus groups until we achieved data saturation, though emergent themes were consistent across the two groups. However, the consistency between our findings and previous work suggests that some of the themes we identified may prove applicable beyond this specific population.

## Conclusion

This study provides much-needed information regarding women’s experiences with anti-nausea medication during pregnancy. Our study and prior studies have shown that many women want and need symptom relief to function at home and work. Women in our study had a wide range of perceptions about the potential risks of these medications. This heterogeneity may be partly due to the lack of rigorous evidence about whether these medications are safe in pregnancy. There is no meaningful information about risks available from randomized trials [9] and there is ongoing debate about the quality and findings of prior observational (epidemiologic) studies [13, 14].

Some women in our study expressed a desire for greater information about risks and benefits of anti-nausea medication use during pregnancy, including about outcomes in diverse populations such as women from different racial and ethnic groups. Future research is needed to clarify the potential risks and effectiveness of widely used anti-nausea medications, including ondansetron, as was highlighted by a recent Cochrane systematic review [9]. More broadly, research is needed to find safe options for managing nausea and vomiting in pregnancy, and this research needs to include women with diverse backgrounds and characteristics.

Our research also sheds light on how women interpret evidence and what kinds of information they find meaningful and compelling. Future qualitative research is needed to understand the ways in which women utilize knowledge and weigh evidence in their decision-making. Such research could help clinicians better understand how to communicate evidence to women in ways that are meaningful and helpful to them.

## Additional file

Additional file 1: Supplementary Material for Reviewer Focus Group Guide. Pregnancy Perspectives - Focus Group Guide: Nausea and Medication Use. This is the guide given to focus group moderators to conduct the conversation for participants. It includes introductory information, questions to pose to the group, and additional probing questions to consider based on elicited conversations. (DOC 44 kb)
