# Supplementary material for: Getting through the day: a pilot qualitative study of U.S. women’s experiences making decisions about anti-nausea medication during pregnancy
Source: BMC Pregnancy Childbirth. 2018 Dec 4;18:475. doi: 10.1186/s12884-018-2093-6 (PMC6280506; doi:10.1186/s12884-018-2093-6)
Supplement: Supplementary file 1 — Supplementary Material for Reviewer Focus Group Guide. Pregnancy Perspectives - Focus Group Guide: Nausea and Medication Use. This is the guide given to focus group moderators to conduct the conversation for participants. It includes introductory information, questions to pose to the group, and additional probing questions to consider based on elicited conversations. (DOC 44 kb) [file 12884_2018_2093_MOESM1_ESM.doc]

**Pregnancy Perspectives**

**Focus Group Guide: Nausea and Medication Use**

**Greet Participants**

**Informed Consent**

**Introduction:**

*Welcome:* Thank you for making time to speak with us today.

*About the project:*

We have invited you here to have a conversation about making decisions during pregnancy. We are interested in learning how women make decisions while pregnant, what matters to them, and what factors they consider. We are especially interested in how women with nausea and/or vomiting in pregnancy (”morning sickness”) make decisions about medication during pregnancy.

There may be many different perspectives on this topic, and that’s okay. In fact, that’s important. Hearing many different thoughts will provide us with a rich understanding of the different perspectives women can have on this topic. Every opinion is valuable, and there are no right or wrong answers.

*This information will be used…*Sometimes pregnant women have to make important choices about their treatments, and sometimes the information they want isn’t here. We will use the information that we learn from you today to inform future research projects. We are interested in conducting research that can help answer questions that are important to women as they make decisions in their pregnancy.
 *Any questions?*

Are there any questions about the purpose of the focus group?

**Content Questions – Nausea and vomiting in Pregnancy**

1. I’d like to begin by inviting you to tell us a little bit about yourself:
   1) What you would like us to call you?
   2) How many children do you have?
   3) What’s one piece of good news you got in the last week?

**Now I would like to ask you about your experience with nausea or vomiting in pregnancy.**

1. We would like to go around and have you briefly describe how nausea during pregnancy affected your life.
   ***Probes:***

- How did it affect your life (at home, and at work)?
- Were there things you would usually do (or wanted to do) that you couldn’t do because of it?
- What bothered you the most about it?
- Did you need extra help from other people at home or at work? Did you get the help you needed?

1. What are some of the things you tried to help you cope with the nausea or vomiting? ***Probe:***

- Did you consider using any natural, herbal or alternative therapies?
  What else did you try?  (ginger, acupuncture, marijuana?)
- Where did you hear about those things?

1. Did your doctor or midwife suggest any treatments? Did he or she offer to prescribe medications to treat your nausea/vomiting? (Vit B6, Unisom)

***Probe:***

- What did your provider tell you about the medications?
- What, if any, risks did they discuss?
- What did they say about how effective the medication was?
- What kind of evidence about effectiveness did they talk about?

1. If you were prescribed a medication, did you fill it? Why did you make the decision to fill it?
   Did you take the prescribed medication? How did you make the decision to take it?

*[verbally note how many raise hands/indicate they actually took the medications]*

1. What was the most important thing that you considered when deciding whether to take a medicine for nausea and/or vomiting in pregnancy?

***Probes***

- Did you consider risks? What risks did you worry about?
- Something happening during the pregnancy itself?
- Fetal development?
- Pregnancy complications? (problems the mom could develop; or problems with the pregnancy such as miscarriage or early labor)
- Longer term effects (such as when the baby is older, maybe age three or four)?

1. Now let’s talk about your experience with medications. For those of you who took medication for nausea, can you describe your experience with medications? [*Note – Ask how many they took, ask questions for each medication*].
   ***Probes:***

- How many times did you take them?
- For how long – how many weeks or months?
- How did they make you feel?
- Did your nausea get better?
- Did you have side effects or problems from the medication? (such as sleepiness)
- Did you have any concerns about the medications after you started taking them?
- Repeat for various medications each woman tried.

1. For this kind of medication, what does effectiveness look like to you? For example, how would you know if an anti-nausea medication was working?
   ***Probes***

- What would be different in your life if it worked well?
- What else would show you that it is effective?

1. Now that you’ve been through all of this, what have you learned? What advice would you give a pregnant friend with nausea who is considering taking these medications?
2. Researchers sometimes study the effects of medications by randomly choosing women to receive certain treatments. Would you be willing to participate in a study that would randomize you to one of several different medications for this symptom? 
   ***Probes:***
   What information would you want to know about the medication before you would agree to participate in such a study?
